# Supplementary material for: From sequence to enzyme mechanism using multi-label machine learning
Source: BMC Bioinformatics. 2014 May 19;15:150. doi: 10.1186/1471-2105-15-150 (PMC4229970; doi:10.1186/1471-2105-15-150)
Supplement: Additional file 2 — Java code of ml2db. Additional file ml2db_code.tar.gz contains the Java source code to run the multi-label machine learning experiments and save the results to database. The code’s Javadoc is included. [file 1471-2105-15-150-S2.zip › additional file 2/ml2db/ml2db/doc/mulan/classifier/meta/ClusteringBased.html]

ClusteringBased


JavaScript is disabled on your browser.


- Overview
- Package
- Class
- Use
- Tree
- Deprecated
- Index
- Help

- Prev Class
- Next Class

- Frames
- No Frames

- All Classes

- Summary:
- Nested |
- Field |
- Constr |
- Method

- Detail:
- Field |
- Constr |
- Method


mulan.classifier.meta

## Class ClusteringBased

- java.lang.Object
- - mulan.classifier.MultiLabelLearnerBase
  - - mulan.classifier.meta.MultiLabelMetaLearner
    - - mulan.classifier.meta.ClusteringBased

- All Implemented Interfaces:
  :   java.io.Serializable, MultiLabelLearner, weka.core.TechnicalInformationHandler

  ---

    

  ```
  public class ClusteringBased
  extends MultiLabelMetaLearner
  ```

  Class implementing clustering-based multi-label classification. For more information, see

  Version:
  :   2012.02.27

  Author:
  :   Gulisong Nasierding, Grigorios Tsoumakas

  See Also:
  :   Serialized Form

- - ### Constructor Summary

    Constructors

    Constructor and Description

    `ClusteringBased()`

    Default constructor.

    `ClusteringBased(weka.clusterers.Clusterer aClusterer,
    MultiLabelLearner aMultiLabelClassifier)`

    Constructor
  - ### Method Summary

    Methods

    | Modifier and Type | Method and Description |
    | --- | --- |
    | `void` | `buildInternal(MultiLabelInstances trainData)` Learner specific implementation of building the model from `MultiLabelInstances` training data set. |
    | `weka.clusterers.Clusterer` | `getClusterer()` Returns the clustering approach |
    | `weka.core.TechnicalInformation` | `getTechnicalInformation()` Returns an instance of a TechnicalInformation object, containing detailed information about the technical background of this class, e.g., paper reference or book this class is based on. |
    | `java.lang.String` | `globalInfo()` Returns a string describing the multi-label learner. |

    - ### Methods inherited from class mulan.classifier.meta.MultiLabelMetaLearner

      `getBaseLearner`
    - ### Methods inherited from class mulan.classifier.MultiLabelLearnerBase

      `build, getDebug, isUpdatable, makeCopy, makePrediction, setDebug`
    - ### Methods inherited from class java.lang.Object

      `equals, getClass, hashCode, notify, notifyAll, toString, wait, wait, wait`

- - ### Constructor Detail


    - #### ClusteringBased

      ```
      public ClusteringBased()
      ```

      Default constructor.


    - #### ClusteringBased

      ```
      public ClusteringBased(weka.clusterers.Clusterer aClusterer,
                     MultiLabelLearner aMultiLabelClassifier)
      ```

      Constructor

      Parameters:
      :   `aClusterer` - the clustering approach
      :   `aMultiLabelClassifier` - the multi-label learner
  - ### Method Detail


    - #### getClusterer

      ```
      public weka.clusterers.Clusterer getClusterer()
      ```

      Returns the clustering approach

      Returns:
      :   the clustering approach


    - #### buildInternal

      ```
      public void buildInternal(MultiLabelInstances trainData)
                         throws java.lang.Exception
      ```

      **Description copied from class: `MultiLabelLearnerBase`**

      Learner specific implementation of building the model from `MultiLabelInstances`
      training data set. This method is called from `MultiLabelLearnerBase.build(MultiLabelInstances)` method,
      where behavior common across all learners is applied.

      Parameters:
      :   `trainData` - the training data set

      Throws:
      :   `java.lang.Exception` - if learner model was not created successfully


    - #### getTechnicalInformation

      ```
      public weka.core.TechnicalInformation getTechnicalInformation()
      ```

      **Description copied from class: `MultiLabelLearnerBase`**

      Returns an instance of a TechnicalInformation object, containing detailed
      information about the technical background of this class, e.g., paper
      reference or book this class is based on.

      **Specified by:**
      :   `getTechnicalInformation` in interface `weka.core.TechnicalInformationHandler`

      **Specified by:**
      :   `getTechnicalInformation` in class `MultiLabelLearnerBase`

      Returns:
      :   the technical information about this class


    - #### globalInfo

      ```
      public java.lang.String globalInfo()
      ```

      **Description copied from class: `MultiLabelLearnerBase`**

      Returns a string describing the multi-label learner.

      **Specified by:**
      :   `globalInfo` in class `MultiLabelLearnerBase`

      Returns:
      :   a description suitable for displaying in a future gui


- Overview
- Package
- Class
- Use
- Tree
- Deprecated
- Index
- Help

- Prev Class
- Next Class

- Frames
- No Frames

- All Classes

- Summary:
- Nested |
- Field |
- Constr |
- Method

- Detail:
- Field |
- Constr |
- Method
